# Aging in Behçet’s syndrome: an overlooked risk for late-life cognitive impairment

**DOI:** 10.1007/s40520-026-03427-y

**Published:** 2026-06-02

**Authors:** Davide Spinetti, Matilde Massara, Camilla Marrone, Elia Salvador, Emma Pepe, Adele Ravelli, Roberto Padoan, Roberta Ramonda, Chiara Ceolin, Giuseppe Sergi, Daniela Mapelli, Marina De Rui, Maria Devita

**Affiliations:** 1 Department of General Psychology, University of Padua, Via Venezia 8, Padua, Italy; 2 Geriatrics Unit, Department of Medicine (DIMED), University of Padua, Padua, Italy; 3 Rheumatology Unit, Department of Medicine (DIMED), University of Padova, Padua, Italy; 4 Department of Neurobiology, Care Sciences and Society, Aging Research Center, Karolinska Institute and Stockholm University, Stockholm, Sweden

**Keywords:** Behçet’s syndrome, Cognitive impairment, Aging, Dementia, Diagnosis, Magnetic resonance imaging

## Abstract

Behçet’s syndrome (BS) is a chronic multisystemic inflammatory disorder with a heterogeneous clinical course. A severe subset with central nervous system involvement (neuro-Behçet’s syndrome, NBS) carries substantial morbidity. While disease activity often decreases with age, the long-term neurological and cognitive outcomes remain poorly defined. Emerging evidence indicates that vascular insufficiency, chronic inflammation, and central nervous system involvement may contribute to cognitive decline in BS, even in the absence of frank neurological symptoms, suggesting a broader and underrecognized impact on brain function. These processes overlap with mechanisms implicated in age-related cognitive decline and dementia, bringing up the hypothesis that BS may accelerate neurodegenerative trajectories in older adults. However, this population remains underrepresented in research. Clarifying whether BS constitutes an overlooked cause or modifier of late-life cognitive decline is clinically urgent. A coordinated agenda spanning neuropsychology, advanced imaging, and biomarkers can deliver diagnostic pathways and testable prevention/management strategies for aging BS populations.

**Supplementary Information:**

The online version contains supplementary material available at 10.1007/s40520-026-03427-y.

## Introduction

Behçet’s syndrome (BS) or Behçet’s disease (BD) is a chronic multisystem inflammatory disorder characterized by a relapsing-remitting course and several clinical manifestations, including oral and genital ulcers, skin lesions, and involvement of ocular, vascular, articular, gastrointestinal, urogenital, pulmonary, and neurologic systems [1]. Although neurological involvement in neuro-Behçet syndrome (NBS) represents one of the most severe disease complications, increasing evidence suggests that cognitive impairment may occur in BS, even in BS individuals without over central nervous system (CNS) involvement [6, 7, 11], suggesting that clinical inflammatory mechanisms may contribute to cognitive decline. Currently, some studies have reported that cognitive dysfunction in BS and NBS affect attention, executive functions, memory, information processing speed and visuospatial abilities [6]. Importantly, different pathophysiological features, such as chronic systemic inflammation, endothelial dysfunction, thrombo-inflammation, vascular insufficiency and immune dysregulation [13–16], as well as psychiatric symptoms and immunosuppressive therapies may further contribute to cognitive decline in this population [6–8]. All these factors overlap with mechanisms known to contribute to age-related cognitive decline and dementia. Given the high mortality rate in younger persons [2], older individuals are currently understudied and underrepresented in BS literature. In fact, current studies involve young or middle aged persons. Due to the potential overlap between the pathological factors of BS and those involved in healthy aging, early diagnosis and appropriate treatment are crucial to minimize long-term neurological complications and improve patient outcomes [1]. On the other hand, the potential long-term neurological and neuropsychological consequences of BS and NBS remain largely unknown. The absence of longitudinal studies investigating dementia risk and long term cognitive trajectories in the BS population are lacking. In this perspective paper we discuss cognitive deficits in BS, epidemiological, inflammatory, vascular and aging-related mechanisms potentially linking BS to progressive cognitive decline, underscoring the important need for longitudinal studies specifically focused on the older BS population.

## Epidemiological evidence linking BD to cognitive impairment and neurodegenerative risk

From an epidemiological perspective, cognitive impairment has been consistently reported in both BS and NBS, affecting individuals with and without overt neurological involvement [3]. Available studies report prevalence estimates ranging from approximately 35% to 50%, with deficits predominantly involving attention, executive functioning, memory, and processing speed [6, 7, 11, 17]. Neuroimaging findings may partially support these observations, as parenchymal NBS lesions involving the diencephalon, brainstem, and basal ganglia have been associated with deficits in attention, executive functioning, and psychomotor speed [5, 7]. In older patients, age-related white matter lesions, cortical atrophy, and microvascular damage may further overlap with BS-related abnormalities, potentially contributing to cognitive vulnerability and complicating neuroradiological interpretation. Current evidence also indicates that disease activity, vascular involvement, chronic systemic inflammation, and longer disease duration may increase the risk of neurocognitive dysfunction, although available studies remain heterogeneous and are largely based on relatively small cohorts. Despite the growing recognition of cognitive impairment in BS, robust epidemiological evidence regarding long-term dementia outcomes remains extremely limited. To date, no large population-based longitudinal studies have specifically quantified the incidence of all-cause dementia, vascular dementia, or Alzheimer’s disease in BS populations. Nevertheless, indirect evidence supporting CNS vulnerability derives from a nationwide Korean cohort study reporting an increased risk of Parkinson’s disease in BS individuals (adjusted hazard ratio 2.47; 95% CI 1.65–3.68) [21], suggesting that chronic inflammation and vasculopathy in BS may contribute to broader neurodegenerative processes. Importantly, most currently available studies involve relatively young or middle-aged patients, whereas older adults (≥ 65 years) remain significantly underrepresented. This represents a major unmet research gap, as aging-related neurodegenerative and vascular mechanisms may synergistically interact with BS-related inflammation and endothelial dysfunction, potentially increasing susceptibility to late-life cognitive impairment and dementia.

## Molecular and cellular mechanisms underlying BD-related cognitive impairment

**Fig. 1:**
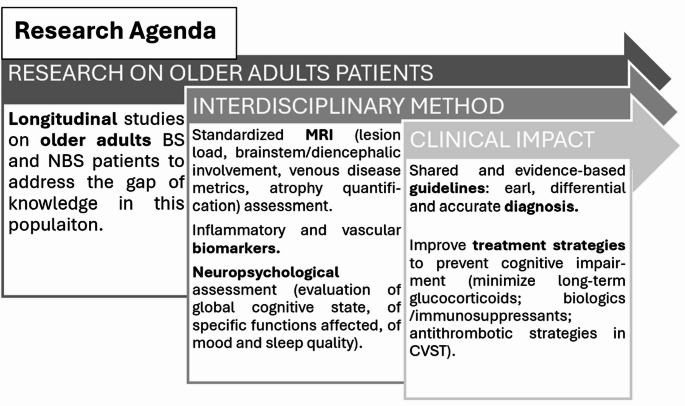
Research agenda

Although the exact etiology of BD has not yet been fully understood, current evidence suggests that the disease arises from aberrant innate and adaptive immune responses triggered by environmental factors in genetically predisposed individuals [14]. Genetic susceptibility factors, particularly HLA-B51 and polymorphisms involving IL-10, IL-23R, and ERAP1, together with infectious triggers such as *Streptococcus sanguis* and herpes simplex virus, may promote chronic systemic inflammation characterized by neutrophil hyperactivity, Th1/Th17 polarization, and increased production of pro-inflammatory cytokines including TNF-α, IL-1β, IL-6, IL-17, and IFN-γ [14, 16]. These inflammatory mediators may contribute to neuroinflammation through endothelial dysfunction and blood-brain barrier disruption, facilitating immune-cell infiltration into the CNS and promoting microglial activation, astrogliosis, oxidative stress, and impaired synaptic plasticity [15]. Persistent neuroinflammation may subsequently contribute to white matter injury, neuronal dysfunction, and disruption of fronto-subcortical networks. In parallel, vascular involvement appears to play a major role in the neurological manifestations of BD [4]. Chronic inflammation promotes endothelial activation, leukocyte recruitment, platelet activation, and a prothrombotic state, ultimately leading to vascular injury and thrombo-inflammation [15]. These mechanisms may contribute to cerebral venous sinus thrombosis, cerebral small vessel disease, chronic hypoperfusion, and ischemic white matter damage, all potentially associated with cognitive decline. Importantly, vascular dysfunction and neuroinflammation likely interact bidirectionally, as endothelial injury may further increase blood-brain barrier permeability and sustain CNS inflammation [13, 15]. Vitamin D has also been shown to modulate inflammatory pathways through inhibitory effects on pro-inflammatory cytokines and NF-κB signaling, mechanisms potentially relevant in chronic inflammatory disorders [18]. Beyond the pathophysiological characteristics, additional factors may further contribute to cognitive dysfunction in BD. Long-term glucocorticoid exposure has been associated with memory impairment, executive dysfunction, and mood alterations, while prolonged immunosuppressive therapies may indirectly affect cognition through fatigue, psychiatric symptoms, or metabolic disturbances [13]. Overall, the interaction among chronic inflammation, neuroimmune dysregulation, endothelial dysfunction, vascular injury, and treatment-related factors likely represents the principal mechanistic framework linking BD to cognitive decline (See Fig. 1, supplementary material).

## Cognitive decline in Behçet and Neuro-Behçet

Cognitive impairment is increasingly recognized as a crucial clinical aspect in BS and NBS. Available evidence suggests a heterogeneous neuropsychological profile, with difficulties reported across executive functioning, attention, information processing speed, memory, visuospatial abilities, and language-related functions. In their systematic review, Fisher & Bernard (2019) [6] found that visuospatial abilities, working memory, and acquired knowledge were the domains most frequently affected, with over 25% of studies reporting significantly poorer performance in both BS and NBS groups compared to healthy controls (HC). In NBS, additional deficits have been described in speed processing and long-term memory encoding and retrieval. Deficits in attention have also been frequently observed, including impaired sustained attention, reduced concentration, slower psychomotor speed, and diminished mental tracking performance, even among BS individuals without overt neurological involvement [11, 22]. Indeed, cognitive dysfunction has been observed also across other BS phenotypes [7]. Cavaco et al. [7] and Fischer & Bernard [6] demonstrated these individuals may perform worse than HC in attention, working memory, delayed memory recall and executive functions, whereas people with NBS exhibited deficits mainly involving attention, learning and executive functions. Table 1 provides a detailed summary of the studies cited above, including the specific cognitive domains assessed and the neuropsychological tests employed. For a more comprehensive and systematic overview of cognitive functioning in BS, readers are referred to the systematic review by Fisher and Bernard (2019) [6]. Collectively, these findings indicate that cognitive impairment in BS and NBS is heterogeneous, multidimensional, and clinically relevant, reinforcing the importance of comprehensive neuropsychological and psychological assessment and early intervention strategies in affected persons.

**Table 1:** Main findings of the cited studies. The cognitive domain showing the most prominent impairment is highlighted, along with detailed information on the specific neuropsychological tests in which significant differences were observed; only tests showing statistically significant group differences are reported

First author (year) and reference	Cognitive Domain most impaired	Neuropsychological Test	Study Design	Sample Size (*n*)	Key Findings
Monastero et al. (2004) [11]	Memory	Rey’s Word-List Learning immediate recall; Rey’s Complex Fig. 10-min recall; Judgment of Line Orientation	Cross- sectional	26 BS 26 HC	Cognitive impairment was observed in 46.1% of BS patients versus none of controls (*p* < 0.0001), predominantly affecting memory (57.7%), with significantly lower performance in immediate verbal recall (t = 3.335, *p* = 0.002), delayed visual recall (t = 6.336, *p* < 0.0001); followed by visuospatial/ constructional abilities (30.8%; t = 3.317, *p* = 0.002), language (19.2%), attention/ psychomotor speed and executive functioning (15.4%) and non-verbal reasoning (3.8%). Impairment was associated with higher disease activity (OR 1.3, 95% CI 1.0-1.5, *p* < 0.04) and higher prednisone dosage (OR 1.3, 95% CI 1.0-1.7, *p* < 0.03).
Gündüz et al. (2012) [23]	Executive Functions and **M**emory	California Verbal Learning Test, Stroop Test, Wisconsin Card Sorting Test, Frontal Behavioral Inventory	Cross- sectional	20 NBS 20 MS	NBS exhibited a significant lower performance in memory and executive functions, characterized by reduced short- and long-term verbal recall, impaired recognition and semantic organization, increased false-positive responses, poorer inhibitory control, reduced cognitive flexibility and greater behavioral disorders.
Ghaleb & Said (2024) [10]	Memory, Attention, Psychomotor Speed	WAIS-R, SCL-90-R, WAIS-R/III	Cross- sectional	40 BS 40 HC	Cognitive impairment was detected in 37.5% of BD patients compared with none of the controls, with memory being the most frequently affected domain (25%), followed by visuospatial processing (20%), language/reasoning/problem-solving and working memory (17.5% each), attention (12.5%), and psychomotor speed (5%). Cognitive functioning was significantly associated with current corticosteroid use and higher depression scores on the SCL-90-R (*p* = 0.01).
Cavaco et al. (2009) [7]	**A**ttention and **S**hort-term memory	Digit Span (WAIS), Corsi Blocks, Stroop, TMT-A TMT-B, Nine-hole peg test	Cross- sectional	15 NBS vs. 32 HC 35 BS vs. 78 HC	Cognitive impairment was detected in 53% of NBS and 40% of BS patients, predominantly involving attention and short-term retention. Compared with HC, NBS patients showed worse cognitive functioning, especially in psychomotor speed (*P* = 0.039) and attention performance (*P* = 0.040), with poorer cognition in NBS was associated with parenchymal involvement. In BS patients lower cognitive performance was related to presence of white matter changes in the frontal lobes, history of headache complaints (especially in attention (*P* = 0.005) and visuospatial memory (*P* = 0.003)), or higher levels of anxiety (with influence in short- (*P* = 0.007) and long-term memory (*P* = 0.020) and attention (*P* = 0.040, *P* = 0.025)), and depression (specifically in attention, *P* = 0.018, *P* = 0.021) .

### Clinical evaluation of cognitive symptoms

As previously reported, many studies have shown cognitive symptoms to be common in BS and NBS, involving multiple cognitive domains [6]. A study by Koc et al. (2026) [19] identified the McNair self-report questionnaire as a useful tool to assess cognitive complaints in parenchymal NBS individuals, as it evaluates multiple cognitive domains (such as attention, concentration, language, temporal orientation, prospective memory, and interpersonal orientation). In addition, standardized instruments such as the Montreal Cognitive Assessment (MoCA), the Symbol Digit Modalities Test (SDMT), the Trail Making Test (A and B), and verbal memory tests have proven to be effective in detecting the pattern of cognitive deficits observed in parenchymal NBS [19]. Nevertheless, a standardized approach for the screening and the assessment of the cognitive status of these individuals has not yet been established, hindering the early diagnosis and identification of BS-related cognitive impairment and the implementation of effective follow-up strategies.

### Clinical impacts of cognitive impairment in daily functioning and quality of life in BS and NBS individuals

Cognitive impairment in BS represents a clinically relevant factor that may interfere with individuals’everyday functioning, psychological well-being, and overall disease burden. Indeed, deficits in attention, memory, executive functioning, and learning abilities, as reported in BS and NBS persons [7, 11], may have meaningful consequences in daily life, particularly for activities requiring planning, prospective memory, problem-solving, and sustained self-monitoring. These abilities are central to instrumental activities of daily living (IADL), including medication management, appointment keeping, financial organization and complex decision-making, and may therefore influence independent functioning and disease self-management [12], ultimately compromising adherence to immunosuppressive therapies and long-term disease monitoring. Additionally, substantial evidence indicates that cognitive dysfunction in BS is frequently associated with psychiatric symptoms, particularly depression, anxiety, and fatigue, which may synergistically worsen functional outcomes and quality of life [6, 8, 11]. Although direct evidence regarding hospitalization rates, institutionalization, caregiver burden, healthcare costs, or mortality remains limited in the current literature in the BS and NBS framework, the persistence of cognitive and psychiatric disorders may plausibly contribute to increased long-term medical and socioeconomic burden. These issues may be particularly relevant in older adults, further emphasizing the importance of early identification and intervention for cognitive dysfunction in this population [12]. Early recognition of cognitive impairment may therefore support more timely multidisciplinary interventions aimed at preserving functional independence, optimizing disease management, and improving long-term outcomes.

## Discussion

Collectively, the findings reported in the present perspective paper suggest that cognitive impairment is frequently observed in both BS and NBS and may progressively worsen over time. Coherently, increasing attention should be directed toward the subset of individuals surviving into older age. BS encompasses not only overt cognitive dysfunction in younger individuals, but also a constellation of inflammatory, vascular, psychiatric, and treatment-related factors that may interact with age-related neurodegenerative mechanisms and potentially increase vulnerability to late-life cognitive decline and dementia. Chronic systemic inflammation, a hallmark of BS, may contribute to neuroinflammation through pro-inflammatory cytokines capable of altering synaptic plasticity, endothelial integrity, and neuronal survival, while simultaneously promoting vascular pathways of cognitive impairment [9]. In parallel, vascular involvement is a core feature of BS, characterized by endothelial dysfunction, thrombo-inflammation, hypercoagulability, and both venous and arterial involvement, including cerebral venous thrombosis [14]. These abnormalities may lead to chronic cerebral hypoperfusion, ischemic white matter injury, oxidative stress, and disruption of fronto-subcortical networks implicated in memory and executive functioning. Importantly, neuroinflammatory and vascular mechanisms likely interact bidirectionally, potentially amplifying neuronal dysfunction and cognitive decline over time. Aging itself may further potentiate these pathological processes through mechanisms of inflammaging, immunosenescence, endothelial senescence, and reduced neurovascular resilience [7, 9, 14, 17]. In older BS population, age-related vulnerability may synergistically interact with chronic systemic inflammation and vasculopathy, potentially exacerbating blood-brain barrier disruption, maladaptive glial activation, cerebral hypoperfusion, and white matter damage. In parallel, aging-associated reductions in neuronal repair mechanisms, autophagic capacity, and cognitive reserve may decrease resilience to chronic neuroinflammatory injury, thereby increasing susceptibility to cognitive impairment and dementia-related processes [20]. These observations support the hypothesis that the interaction between biological aging and BS-related inflammatory-vascular pathology may represent a critical upstream driver of late-life cognitive decline. Several additional factors may further confound the relationship between BS and cognitive impairment. Traditional cardiovascular risk factors, including hypertension, diabetes mellitus, dyslipidemia, and smoking, may coexist with systemic vascular inflammation and independently increase dementia risk. Psychiatric manifestations such as depression, anxiety, chronic fatigue, and psychological distress are also highly prevalent in BS and may impair cognitive performance independently of structural neurological damage [17]. Moreover, psychiatric and cognitive symptoms may occur even in the absence of overt MRI abnormalities, suggesting that functional and neuroimmune mechanisms may substantially contribute to cognitive complaints. Long-term exposure to glucocorticoids and immunosuppressive therapies may additionally influence cognition through mood alterations, fatigue, metabolic disturbances, or potential neurotoxic effects [14, 17]. Socioeconomic status, educational attainment, and health literacy may further affect the recognition and management of cognitive impairment, potentially contributing to underdiagnosis and heterogeneity across studies. To conclude, future longitudinal studies should systematically control for vascular risk factors, psychiatric comorbidities, treatment exposure, and socioeconomic variables in order to better clarify whether BS independently contributes to cognitive decline and dementia risk. To date, no studies have specifically characterized cognitive functioning in older adults with BS or NBS. Consequently, whether BS should be conceptualized as a potential contributor to vascular dementia, a condition interacting with neurodegenerative processes, or a distinct inflammatory-neurovascular model of cognitive decline remains unresolved and warrants systematic investigation (See Research Agenda, Fig. 1).

## Conclusions

This perspective article underscored the underexplored aspects of neurological and cognitive manifestations in BS, particularly in older adults. We discussed how the potential interplay between disease features and age-related mechanisms may contribute to progressive cognitive decline and potentially increase dementia risk in older BS individuals. Given the limited number of epidemiological and longitudinal studies currently available, future studies targeting older population with BS are needed to better understand cognitive profile and its trajectory over time. This will allow also to identify BS-related risk factors, and develop standardized strategies for early diagnosis and multidisciplinary management.

## Supplementary Material

Below is the link to the electronic supplementary material.

Supplementary Material 1

## Data Availability

No datasets were generated or analysed during the current study.
