# Supplementary material for: Aging in Behçet’s syndrome: an overlooked risk for late-life cognitive impairment
Source: Aging Clin Exp Res. 2026 Jun 2;38(1):172. doi: 10.1007/s40520-026-03427-y (PMC13442495; doi:10.1007/s40520-026-03427-y)
Supplement: Supplementary file 1 [file 40520_2026_3427_MOESM1_ESM.docx]

Supplementary material
